# Effect of Water-Saving Society Policy on Water Consumption in the Cities of China: A Propensity Score Matching Analysis

**DOI:** 10.3390/ijerph17218171

**Published:** 2020-11-05

**Authors:** Yali Zhao, Min Li

**Affiliations:** 1 School of Public Administration, Hohai University, Nanjing 211100, China; 2 College of Public Administration, Nanjing Agricultural University, Nanjing 210095, China; 2019209029@njau.edu.cn

**Keywords:** water-saving society policy, water consumption, propensity score matching, treatment effect, water scarcity

## Abstract

The increased demand for water resources due to urban population and economic growth has worsened the urban water crisis. In order to address this issue, a policy of “developing a water-saving society” (namely, water-saving society policy) has been implemented in some Chinese cities. This study takes 285 cities at the prefecture level and above as the sample and uses the propensity score matching (PSM) method to analyze the effect of China’s urban water-saving society policy on the reduction of water consumption per CNY 10,000 gross domestic product (GDP) from 2005 to 2017. The results show that the water-saving society policy significantly (*p* < 0.01) reduced water consumption in the study period; however, the effects differed between cities with different water resource endowments, economic development level, and urban scale. Specifically, there was a positive water consumption reduction effect in cities in humid areas, with low economic development, or of large scale, while the effect was limited in cities in arid areas, with high economic development, or of small scale. Therefore, for areas where water resource supply is insufficient, water-saving policy should be designed and implemented suiting local conditions, and it is also necessary to explore more water sources.

## 1. Introduction

Water is one of the most important natural resources, which supports not only ecosystem sustainability but also the development of almost all sectors [1]. However, as water demand continues to increase and reaches its peak, human water use is beyond sustainable levels throughout the world [2]. Due to the irreplaceability of water resources in the development of cities, rapid population growth, fast urbanization, increasing economic development, unprecedented technological innovations, drastic land-cover alternations, and climate change have inevitably led to a rapid growth in urban water demand and a severe water supply crisis [3,4]. In some particular industries, such as iron and steel industry, tremendous contributions to their rapid development are highly reliant on the intensive water consumption [5,6]. Besides high water consumption industries, households are also the main consumers of water resources [7]. Psychosocial and behavioral factors affect the intention of consumers to carry out water-saving actions [8,9], causing differences in water consumption between households. Additionally, large amounts of fresh water, soil water, and related virtual water “trade” are playing vital roles in the sufficient supply of food [10,11]. Competition for water and the balance between supply and demand for food are also widely concerned [12,13,14]. Compared with the growing water demand, the supply of water from traditional sources is unsatisfactory, due to environmental constraints, storage capacity expansion limitation and extraction restrictions [15], the tensions between water reallocation from rural to urban areas [16,17,18], etc. At present, scholars are paying attention to alternatives, such as rainwater catchment systems, to supplement water sources [19,20,21]. However, rainwater is easily affected by climate change; water demand and water resource availability are affected by climatic conditions changes [21], which exacerbate the vulnerability of water resources [22]. As the diverse water nexus makes water management complicated, benefit balancing and institutional arrangements are therefore adopted to resolve water-use conflicts [23,24].

Water saving has been widely regarded as one of the most effective ways to reduce water scarcity. Urban water-saving projects can improve water-use efficiency and reduce water waste in various ways; for example, economic incentives (e.g., water pricing), technical improvements (e.g., water-saving household appliances), or regulations can increase domestic water savings [25]. Both the Chinese and global experiences have shown that there is a positive effect of water-saving measures on urban water consumption reduction [26,27,28]. However, water-saving measures only restrain the growth rate of water consumption; the total water consumption is still increasing as the population and economy continue to grow [29,30,31]. It is more critical that the water consumption reduction from urban producers and consumers in water saving can be offset by increasing virtual water exports in goods and services to a large extent [32]. As a result, the improvement of water-use efficiency and water technology will not necessarily reduce the total water consumption [33,34,35]. In addition, there is a significantly negative correlation between water resource endowment and water-use efficiency: the more abundant the water resources are, the more extensive the water resource use is [36]. Thus, water-saving operations need huge governmental subsidies to ensure water-saving effects [37]; however, water-saving subsidies may actually increase regional water consumption under some circumstances [38]. Taking the external environment into account, water-saving and conservation technologies may have detrimental impacts on environmental flows in certain circumstances [39,40]. Therefore, water saving is a complex issue involving multiple factors, and the effects of water-saving policies need to be further studied.

China is faced with resource shortage and pollution issues [41], being one of the countries with the lowest water resources per capita in the world. The total water resource consumption in China keeps increasing annually [42]. Previous research has shown that the amount of available water resources, the domestic water price, and the usage rate of water-saving appliances are critical factors affecting the daily water consumption per capita in China [43]. In order to improve the sustainability of water resources, the Chinese government has adopted a policy to develop a “water-saving society” (namely water-saving society policy), as incorporated into the revised Water Law of China in 2002 [32]. In the same year, the Ministry of Water Resources of China made an announcement regarding launching a pilot project for building a water-saving society, and then a series of cities successively implemented the policy in the period of 2002–2005, and the years of 2006, 2008, and 2010. As the water-saving society policy was implemented, effects of the policy such as water-use efficiency [44], water consumption [32,45], and comprehensive performance [46] in pilot cities were examined; the logical framework and the index evaluation [44], structural decomposition analysis approach [32], matter element analysis method [45], and AHP (Analytic Hierarchy Process)-fuzzy comprehensive evaluation model [46] were employed. However, during the water-saving society policy implementation period, factors such as simultaneous water-saving technology upgrades and other policies may have also taken place. We thus have to adopt a reliable method to eliminate the impacts of other simultaneous factors for revealing the effect of urban water-saving society policy.

To better understand the relationship between water-saving society policy and water consumption, we attempt to address the following questions: To what extent does the water-saving society policy affect urban water consumption? Do any differences exist between the effects of different cities? We then take water consumption per CNY 10,000 GDP (m^3^/(CNY 10^4^ ), USD 1 ≈ CNY 7) as a measure and report the work as follows: Section 2 describes the method, study area, and data; Section 3 presents the results; Section 4 discusses reasons for the observed effects in different situations; and Section 5 concludes.

## 2. Method and Materials

### 2.1. Propensity Score Matching Method

Remote sensing and geographical information system analysis are often adopted to examine the effect of human activities on water resources or landscape change [47,48]. Although combined with post-classification comparison, sudden change caused by immediate factors like extension of roads and infrastructure or critical political events can be clearly explored [48], impacts of other simultaneous factors should be further considered. Our goal in this study is to estimate the effect of urban water-saving society policy on water consumption. The effect of an implemented policy is usually called the treatment effect in economics. For each city *i*, *i =* 1, 2, 3, …, N, there will be two potential water consumption outcomes {*Y*_*i*0_, *Y*_*i*1_}. *Y_i0_* represents the water consumption of the city without implementing the water-saving society policy; *Y*_*i*1_ represents the water consumption of the city which implements the water-saving society policy. Accordingly, cities implementing the water-saving society policy are classified as a treated group (*D_i_* = 1), and cities without implementing the policy are classified as the control group (*D_i_* = 0). To evaluate the effect of a city’s water-saving society policy, the best situation is to be able to simultaneously obtain the water consumption data of a city implementing and without implementing a water-saving society policy during the study period, where the treatment effect of the water-saving society policy is *Y*_*i*1_ − *Y*_*i*0_. For the full sample, the average treatment effect (ATE) can be defined as

(1) $$\mathrm{ATE}=E\left(Y_{i1}-Y_{i0}\right)=\frac{1}{M}\overset{M}{\underset{i=1}{\sum }}\left(Y_{i1}-Y_{i0}\right),$$

where *M* is the sample size. As water consumption is a value of water-use outcome, E (*Y*_*i*1_ − *Y*_*i*0_) can be estimated by directly comparing water consumption outcomes and does not need to use a functional form or impose any distribution. However, during a certain study period, only one state of a city can be observed—that is, water consumption when the policy has been implemented or not implemented—not the two states at the same time. This is called “counterfactual missing data”. Following the counterfactual framework of Rosenbaum and Rubin (1983) [49], the average treatment effect of cities implementing the water-saving society policy, namely the average treatment effect on the treated (ATT), can be defined as:

(2) $$\mathrm{ATT}=E(Y_{i1}-Y_{i0}|D_{i}=1)=E\left(Y_{i1}|D_{i}=1\right)-E\left(Y_{i0}|D_{i}=1\right)$$

where ATT indicates the difference in water consumption between the cities implementing a water-saving society policy under the assumption that they have not yet. Obviously, E(*Y*_*i*0_|*D_i_* = 1) cannot be observed. In addition, whether a city implements a water-saving society policy or not is related to the city’s economic, social, and water resource conditions; it is not implemented randomly. Therefore, the calculation method based on the assumption of random samples will lead to biased results. To solve these problems, this study adopts the propensity score matching (PSM) method, which solves the problem of selective bias in non-random sample data by constructing a counterfactual framework [49,50], to evaluate the ATT.

Following Rosenbaum and Rubin (1983) [49], we need to match the cities of the control group and the treated group according to their propensity scores; that is, we must find city *j* in the control group which matches the characteristic variables (*X*) of city *i* in the treated group as similarly as possible, namely *Xi* ≈ *Xj*. Then, the water consumption E(*Y_i0_*|*D_i_* = 0) of the control group can be regarded as the water consumption E(*Y_i0_*|*D_i_* = 1) of the cities in the matched treated group, which is equivalent to “simultaneously” observing the water consumption of implementing and not implementing the water-saving society policy. Specifically, the propensity score for this study is the city’s probability of implementing the water-saving society policy, conditional upon covariates. As *D_i_* = 1 indicates that the city implements the water-saving society policy and 0 otherwise, the propensity score can be expressed as [51],

(3) $$P\left(X\right)=Logit\left(D_{i}=1|X\right)$$

where *P*(*X*) is the propensity score and *X* is multiple characteristic variables of cities that affect the probability of implementing the water-saving society policy. As the implementation of urban water-saving projects is mainly related to the urban population, water resources, and economic development, we selected population density (*X*_1_, person/km^2^), GDP per capita (*X*_2_, CNY per capita), ratio of the secondary industry output to the tertiary industry output (*X*_3_, dimensionless unit), centralized treatment rate of urban sewage (*X*_4_, %), and water supply per capita (*X*_5_, m^3^ per capita) as characteristic variables.

Please note that before propensity score matching, some assumptions such as common support, balancing assumption, ignorability and so on should be considered [49]. We first considered the assumption of common support, which guarantees the overlap of the propensity scores between the treated group and the control group (that is, the two groups have similar characteristics) and the balancing assumption, which means the *X* characteristics of the treated group and the control group are similar. If the two assumptions hold, E(*Y*_*i*0_|*D_i_* = 1) can be replaced by E(*Y*_*i*0_|*D_i_* = 0) approximately in formula (2), and ATT can be further estimated as follows:

(4) $$\mathrm{ATT}=E\left(Y_{i1}|D_{i}=1\right)-E\left(Y_{i0}|D_{i}=0\right)=E\left(Y_{i1}|D_{i}=1,\;P\left(X\right)\right)-E(Y_{i0}|D_{i}=0,P\left(X\right))$$

In Formula (4), the methods used to match samples between the treated and control groups included nearest neighbor matching (NNM), radius matching (RM), kernel matching (KM), local linear regression matching (LLRM), and others. In this study, various matching methods were adopted, and the results were compared. If the ATTs of different methods were similar, the effect of water-saving society policy on water consumption was considered to be robust. As the PSM is a nonparametric estimator, once a matched sample has been formed, the ATT would be estimated by directly comparing water consumptions between the treated and control groups.

Finally, the PSM analysis can be realized through the “psmatch2” order of Stata 16.0 (StataCorp, College Station, TX, USA), and the basic format is psmatch2 D *X*_1_ *X*_2_ *X*_3_ *X*_4_ *X*_5_ outcome(*Y*) ties logit common. The balancing assumption test and common support assumption test can also be made by the “pstest” and the “sum _pscore” order of Stata 16.0, respectively.

### 2.2. Study Area

**Figure 1:**
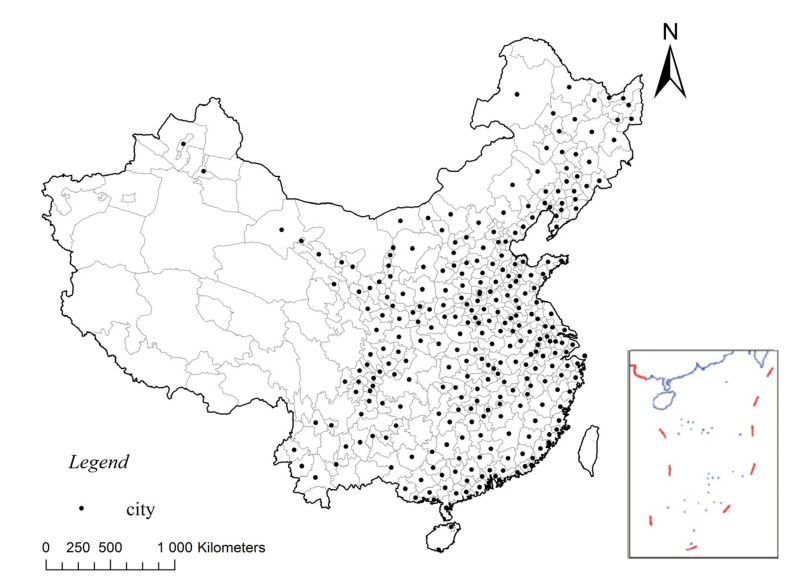
Location of the study area.

Due to rapid urbanization and administrative adjustment in China, the number of cities frequently changed in the past several decades [52]. To obtain a representative and fixed sample, we selected 285 cities at the prefecture level and above as the study area (Figure 1). In accordance with Riad et al. [48], cities differing in water endowment, economy, and size were included.

A city was marked as 1 (D = 1, treated group) after implementing the water-saving society policy, or 0 (D = 0, control group) otherwise. Considering the vast territory of China, not only all sampled cities but also parts of cities with the similar characteristics of water resource endowment, urban economic development, and city scale should be examined. That is, the full sampled cities were classified into several groups as follows:

(1) City classification based on the annual precipitation in the area where the city is located, namely, cities in arid areas, where the annual precipitation was less than 200 mm and the amount of water that can be stored and used is small; cities in humid areas, where the annual precipitation was greater than 800 mm and the available water resources are sufficient; and cities in semi-humid or semi-arid areas, where the annual precipitation was between 200 mm and 800 mm.
(2) City classification based on the per capita GDP of each city, namely, high economic development cities (with per capita GDP higher than the national average urban per capita GDP) and low economic development cities (with per capita GDP lower than or equal to the national average urban per capita GDP).
(3) City classification based on the Chinese city scale classification standard during the study period, namely, small cities (with a resident population of less than 500,000); medium cities (with a resident population of more than 500,000 and less than 1 million); and big cities (cities with a resident population of more than 1 million).

### 2.3. Data

Considering data availability and policy implementation, the starting point of the evaluation was 2005, and mixed cross-sectional data of 285 cities in China from 2005 to 2017 were constructed. The GDP, total water consumption, total water supply, GDP per capita, population density, urban population, the proportion of the secondary industry, the proportion of the tertiary industry, and the centralized treatment rate of urban sewage of each city were obtained from the *China City Statistical Yearbook* [53] and the *China Urban Construction Statistical Yearbook* [54]. However, the population densities in 2016 and 2017 were not given in the statistical yearbook, and, thus, the ratio of population to city area was used for calculation instead. The per capita water supply was calculated by the ratio of total urban water supply to urban population. GDP and per capita GDP were converted into constant prices of 2005 using GDP deflation and per capita GDP deflation, respectively; namely, constant-price GDP*_t_* = nominal GDP*_t_*/GDP deflation, GDP deflation = GDPI_2005_ × GDPI_2006_ × … × GDPI*_t_*, GDPI is GDP index/100, and GDPI_2005_ = 1, where *t* indicates the year. GDP indices were obtained from the *China Statistical Yearbook* [55]. Constant-price per capita GDP was calculated by the same method. In addition, because some cities lacked data for some years, we finally obtained 3412 records of the sampled cities for the PSM analysis (see Supplementary Material Tables S1 and S2).

## 3. Results

### 3.1. Balancing Assumption and Common Support Test Results

In order to obtain a perfect matching effect, the balancing assumption was first tested based on various matching methods of NNM, RM, KM, and LLRM that can be used to match samples. From Table 1, most of the matched mean biases were less than 10% and reduced when compared to the unmatched equivalents. Although the *p*-values of *X_2_* were all significant, most of the % biases were not significantly different from zero after matching, and this means that there were no significant differences between the treated group and control group after samples were matched. It can be inferred that the distribution of the two groups is balanced in general.

Then, the common support assumption was examined. From Table 2, cities implementing water-saving society policy have propensity scores ranging between 0.043 and 0.828, and ranging between 0.039 and 0.999 otherwise. Hence, the region of common support lies within a propensity score of 0.043 and 0.828, and only samples whose propensity scores lie within this region were used in this study. Therefore, the PSM analysis and NNM, RM, KM, and LLRM are all proper.

### 3.2. The Effect of Water-Saving Society Policy on Water Consumption

#### 3.2.1. Urban Water-Saving Society Policy Significantly Reduced Water Consumption of Cities in China during the Study Period

From Table 3, under full-sample conditions, the AATs of the water-saving society policy on water consumption per CNY 10,000 GDP were significantly negative at the level of 1% and were between −9.17 and −8.28 (i.e., roughly the same) under the different matching methods. That is, after the implementation of the urban water-saving society policy, the water consumption decreased by 8.28~9.17 m^3^/(CNY 10^4^) compared to without the policy. The results showed that the water-saving society policy worked effectively in the study area and period.

#### 3.2.2. The Effect in Humid Areas, Semi-Humid and Semi-Arid Areas, and Arid Areas Decreases Successively

From Table 4, the ATTs in cities in humid areas, as well as semi-arid or semi-humid areas, on water consumption were generally significantly negative, indicating that the water consumption had decreased significantly (*p* < 0.01) after the implementation of the water-saving society policy in these areas. At the same time, the AATs of cities in humid regions (more than m^3^/(CNY 10^4^)) were much higher than that of cities in semi-arid and semi-humid areas (less than 5.05 m^3^/(CNY 10^4^)). The AATs in arid areas were not significant, which indicate that the water-saving society policy had not worked effectively in these areas. It can be observed that there were differences in the effects of the urban water-saving society policy in areas with different water resource endowments. Its effect in cities in humid areas was the most significant (*p* < 0.01), followed by cities in semi-arid and semi-humid areas, while its effect in cities in arid areas was not significant.

#### 3.2.3. The Effect in Low Economic Development Cities Is More Significant and More Stable than in High Economic Development Cities

From Table 5, the water-saving society policy in cities with diverse economic development had a negative average treatment effect on water consumption. According to the ATT values that passed the significance test, after the implementation of the urban water-saving society policy, the water consumption decreased by 1.87~3.13 m^3^/(CNY 10^4^) in cities with high economic development, and it is significant at the 10% level, while the water consumption decreased by 2.80~3.55 m^3^/(CNY 10^4^) with a significance at the 1% level in low economic development cities. It can be seen that the water consumption reduction effect in low economic development cities was more significant, sizable, and stable than that in high economic development cities.

#### 3.2.4. The Effect in Big- and Medium-Sized Cities Is Significant, but Not in Small Cities

From Table 6, the effect in big cities was significantly negative (*p* < 0.01) and the results (decreased 3.54~4.34 m^3^/(CNY 10^4^)) were relatively stable, while the effect in medium-sized cities was also negative (decreased 5.33~6.95 m^3^/(CNY 10^4^)), but the significance was lower and less stable than that of big cities. The ATTs in small cities were negative, but the effect was smaller than that of both big- and medium-sized cities, and it did not pass the significance test. Hence, the water-saving society policy significantly reduced water consumption in big cities, while that of medium-sized cities decreased to a certain extent, and, in small cities, it did not significantly decrease.

## 4. Discussion

There are two major ways to solve water resource problems: one is improving the water resource infrastructure by hardware construction, and the other is improving systems, adopting high-efficiency technologies, and protecting the environment through software construction [56]. To achieve the goal of urban water conservation, it is important to improve water production capacity and reduce water loss in production and transportation through the construction of water resource-use infrastructure [57,58], such as urban water source protection projects, water purification and storage facilities and water transportation pipelines. As water scarcity variation has mainly been related to the properties of water and its role in natural–human systems [59], attention should also be paid to scientific incentive mechanisms for urban water-use through the innovation and promotion of water-use technologies [60], rights transactions [61], pricing [62,63], and the cultivation of water-saving awareness [9]. It is undoubted that the water-saving society policy in China can effectively reduce water consumption per CNY 10,000 GDP, as mentioned above, by increasing water-saving investments and developing water-saving behaviors. However, it should also be noted that the external environment for implementing the water-saving society policy differed per city, which featured different water resource endowments, economic development levels, and city scales. The reasons for the observed effects of the water-saving society policy in different situations are further discussed in the following subsection.

### 4.1. Water Resource Endowment and the Effect of Water-Saving Society Policy

In the situation of a shortage of natural supply of urban water resources and water for production and living, urban residents deeply understand the inconvenience caused by water shortages and the importance and urgency of water-saving hardware and software construction. Previous research has found that the attitudes of stakeholders towards water-saving policies have positive and significant effects on water-saving behaviors [64,65]. Water-saving policies are also generally implemented first in water shortage areas. As a result, the urban water resource endowments in arid areas are insufficient, the concept of water-saving has already been deeply rooted in the hearts of people, the construction of water-saving hardware and software has been promoted earlier, and the space for the water-saving policy to play a role is relatively limited, such that it is difficult to reduce water consumption by a large amount. In contrast, cities in humid regions have sufficient natural endowments of water resources, and, thus, water conservation is not considered urgent. In these cities, water consumption has been large for a long time, and there is ample room for the water-saving society policy to play a vital role in reducing water consumption. This explains why the aforementioned urban water-saving society policy had significant effects in humid areas, while its effect in arid areas was not significant.

### 4.2. Economic Development and the Effect of Water-Saving Society Policy

Economic development determines the capital, technology, and other resources that can be used in urban water conservation. When a city’s economic development is low, there is no corresponding capital or technical capacity to invest in water-saving hardware and software, making it impossible to achieve the goal of alleviating water scarcity. When urban economic development reaches a high level, the demand of urban water resources tends to be stable, and the improvement in urban economy, technology, and external environmental conditions can strongly support the construction of water-saving infrastructure and software. Accordingly, although the implementation of the water-saving society policy will improve the capital, technological, and systematic conditions of these cities, the water-saving effect on cities differed along with their economic development. For cities with low economic development, there is more water-saving space due to formerly poor water-saving practices, such that the policy will work well; otherwise, cities with high economic development have less water-saving space, and the policy effect is limited. This explains the observed difference of the average treatment effect of the water-saving society policy in cities with different economic development conditions.

### 4.3. City Scale and the Effect of Water-Saving Society Policy

As scales become arenas for power performing and policy making [66,67], and institutional arrangements would be framed by a main scale [68], the city scale should thus be considered to understand the effect of urban water-saving policy. In general, big cities in China have a good industrial foundation, human capital, technology promotion, and water-saving systems. Water-saving society policy implementation can speed up infrastructure construction (e.g., water purification and storage facilities, transportation pipelines, etc.), eliminate industries with high water consumption, change water-use behaviors of residents and enterprises, reduce water waste, improve water-use efficiency, and, finally, achieve water-saving effects. However, small cities are not attractive to new industries and technology upgrade on one hand, and with poor water-saving practices and systems on the other. The traditional industries in small cities are difficult to be replaced, and small cities may even undertake high water-consuming industries transferred from big cities. Taken population, material and institutional conditions of cities at different scales into consideration, the water-saving society policy cannot change the traditional water-use situation and cannot lead to a scale effect of water-saving in small cities. To a certain extent, this reveals the reasons for the differences in the effect of the water-saving society policy in cities of different sizes.

## 5. Conclusions

Propensity score matching analysis is a useful tool to eliminate simultaneous factors’ impacts in evaluating the effect of an implemented policy. Based on this method, the study demonstrates the positive effect of the water-saving society policy on the reduction of water consumption from 2005 to 2017 in Chinese cities. However, differences existed between cities with different water resource endowment, economic development level, and urban scale. In general, the effect was significant in humid areas, cities with low economic development, and big cities, while not in arid areas, cities with high economic development, and small cities. The effect was also closely related to the water-saving capacity, institutional arrangements and external environment for the water-saving society policy implementation. It can thus be inferred that in order to realize sustainable water use of cities and to achieve the goal of water consumption reduction, water-saving policy should be designed and implemented suiting local conditions.

In addition, although demand-side water management policies may be substantially more effective than supply-side approaches [69], this study also showed that, for areas where the natural supply of water resources is absolutely insufficient, water shortages cannot be solved by relying on water-saving measures alone. It is important to reallocate water resources across different areas in the future. An alternative approach is to transfer the water saved in water-rich areas to water-scarce areas; otherwise, the effect of the water-saving policy will be difficult to transform into the water-saving effectiveness of the whole society. The problem faced by this approach is that it not only costs a lot of money and involves a long construction period, but may also lead to serious social and ecological consequences [56]. Cross-regional allocation of water resources must be adapted with an operational arrangement for water cooperation [70,71] and overcoming barriers related to institutional environment and path dependencies [72], social-environmental justice [73] and political challenges [67,68,74]. Another option is to reduce the reverse flow of water from water-scarce areas to water-rich areas caused by virtual water transfer, mainly occurring through internal trade [75]. To achieve this objective, incentive mechanisms for promoting water-saving technologies in industry [75], technical cooperation and technology transfer [23], and optimizing urban industries distribution between water-scarce and water-rich areas are available measures.

Furthermore, the construction of urban water conservation infrastructure must be carried out as soon as possible and unremittingly for a long time. With economic and social development and the associated increasing water demands, cities will eventually face the production and living difficulties caused by water shortages. If the water-saving society policy is regarded as a tool to solve the demand side of water resources, then exploring more available water sources is an important way to solve the problem of water on the supply side. Only by adjusting both the supply side and demand side of urban water resources at the same time can the problem of urban water resources be better solved. The results and recommendations are meaningful and contribute to the development of a water-saving society in China and other countries’ cities.

## Figures and Tables

**Table 1:** Variable balance between treated and control groups, before and after matching.

Variable	U/M	NNM		RM		KM		LLRM	
		%bias	*p*-Value	%Bias	*p*-Value	%Bias	*p*-Value	%Bias	*p*-Value
*X*_1_	U	25.9	0.000	25.9	0.000	25.9	0.000	25.9	0.000
	M	−13.8	0.051	−6.9	0.215	−5.7	0.364	−13.8	0.051
*X*_2_	U	22.1	0.000	22.1	0.000	22.1	0.000	22.1	0.000
	M	8.6	0.004	7.1	0.015	9.0	0.002	8.6	0.004
*X*_3_	U	−5.6	0.171	−5.6	0.171	−5.6	0.171	−5.6	0.171
	M	−6.5	0.176	−1.7	0.721	−3.0	0.536	-6.5	0.176
*X*_4_	U	45.0	0.000	45.0	0.000	45.0	0.000	45.0	0.000
	M	0.7	0.874	−0.3	0.944	1.1	0.796	0.7	0.874
*X*_5_	U	19.4	0.000	19.4	0.000	19.4	0.000	19.4	0.000
	M	−3.1	0.593	−2.2	0.696	−1.2	0.836	−3.1	0.593

Notes: U = unmatched and M = matched. *X*_1_ = population density, *X*_2_ = GDP per capita, *X*_3_ = ratio of the secondary industry output to the tertiary industry output, *X*_4_ = centralized treatment rate of urban sewage, *X*_5_ = water supply per capita. NNM = nearest neighbor matching, RM = radius matching, KM = kernel matching, LLRM = local linear regression matching. %bias means standard mean %bias. The null hypothesis is that the %biases are not significantly different for any variable in the treated and control groups.

**Table 2:** Propensity score distribution for treated and control cities.

Propensity Score for Cities	Mean	Min	Max	Std.Dev.	Obs
Implementing water-saving society policy	0.278	0.043	0.828	0.103	800
Without implementing water-saving society policy	0.221	0.039	0.999	0.090	2612

**Table 3:** Average treatment effect on the treated (ATT) estimates under full-sample conditions.

Category	Matching Method	Treated	Control	ATT	Std.	T.
All cities	NNM	34.24	43.41	−9.17 ***	1.77	−5.16
	RM	34.17	42.46	−8.28 ***	0.95	−8.71
	KM	34.24	42.69	−8.44 ***	0.93	−9.08
	LLRM	34.24	43.01	−8.77 ***	1.77	−4.93

Notes: *** Statistical significance at 1% level. Treated = treated group, namely cities implementing the water-saving society policy; Control = control group, namely cities without implementing the policy. The meaning of headings of Table 4, Table 5 and Table 6 is the same as Table 3.

**Table 4:** ATT estimates in cities with different water resource endowments.

Category	Match Method	Treated	Control	ATT	Std.	T.
Cities in arid areas	NNM	33.33	34.39	−1.05	5.25	−0.20
	RM	34.54	35.30	−0.75	4.62	−0.16
	KM	33.33	35.24	−1.91	5.56	−0.34
	LLRM	33.33	36.46	−3.13	5.25	−0.60
Cities in semi-humid or semi-arid areas	NNM	31.32	36.38	−5.05 ***	1.70	−2.96
	RM	31.32	36.06	−4.74 ***	1.09	−4.33
	KM	31.32	36.16	−4.83 ***	1.07	−4.52
	LLRM	31.32	35.42	−4.09 **	1.70	−2.40
Cities in humid areas	NNM	38.35	55.98	−17.63 ***	3.42	−5.15
	RM	37.66	45.79	−8.12 ***	1.44	−5.61
	KM	38.35	54.84	−16.49 ***	1.60	−10.26
	LLRM	38.35	53.21	−14.85 ***	3.42	−4.34

Notes: ** Statistical significance at 5% level; *** statistical significance at 1% level.

**Table 5:** ATT estimates in cities with different economic development.

Category	Match Method	Treated	Control	ATT	Std.	T.
Cities with high economic development	NNM	31.15	34.29	−3.13 *	1.81	−1.73
	RM	29.87	31.74	−1.87 *	1.01	−1.84
	KM	31.00	33.20	−2.20 *	1.28	−1.71
	LLRM	31.15	31.89	−0.73	1.81	−0.40
Cities with low economic development	NNM	35.87	37.65	−1.78	1.32	−1.34
	RM	35.92	38.73	−2.80 ***	1.06	-2.63
	KM	35.87	39.42	−3.55 ***	1.04	−3.39
	LLRM	35.87	39.01	−3.14 ***	1.32	−2.36

Notes: * Statistical significance at 10% level; *** statistical significance at 1% level.

**Table 6:** ATT estimates in cities of different sizes.

Category	Match Method	Treated	Control	ATT	Std.	T.
Small cities	NNM	29.46	31.57	−2.11	3.35	−0.63
	RM	30.75	33.01	−2.25	2.76	−0.82
	KM	29.79	32.17	−2.38	2.76	−0.86
	LLRM	29.46	32.02	−2.55	3.35	−0.76
Medium-sized cities	NNM	38.34	44.09	−5.74 *	3.20	−1.79
	RM	38.18	43.52	−5.33 ***	1.84	−2.89
	KM	38.18	45.14	−6.95 ***	1.78	−3.89
	LLRM	38.34	43.32	−4.97	3.20	−1.55
Big cities	NNM	33.72	38.07	−4.34 ***	1.56	−2.77
	RM	33.13	36.67	−3.54 ***	1.09	−3.25
	KM	33.66	37.38	−3.71 ***	1.22	−3.03
	LLRM	33.72	37.89	−4.17 ***	1.56	−2.66

Notes: * Statistical significance at 10% level; *** statistical significance at 1% level.

## Supplementary Materials

The following are available online at https://www.mdpi.com/1660-4601/17/21/8171/s1, Table S1: data, Table S2: variable definition.
